# Beyond Cat Scratch Disease: A Case Report of *Bartonella* Infection Mimicking Vasculitic Disorder

**DOI:** 10.1155/2012/354625

**Published:** 2012-08-08

**Authors:** Amelia Spinella, Federica Lumetti, Gilda Sandri, Valentina Cestelli, Maria Teresa Mascia

**Affiliations:** ^1^Rheumatology Unit, Department of Internal Medicine, University of Modena and Reggio Emilia, Via del Pozzo 71, 41124 Modena, Italy; ^2^Unit of Malattie dell'Apparato Locomotore a Genesi Immunologica, University of Modena and Reggio Emilia, Via del Pozzo 71, 41124 Modena, Italy

## Abstract

Cat scratch disease (CSD) is a bacterial disease caused by *Bartonella henselae* and it is mainly characterized by self-limiting lymphadenopathy in the draining site of a cat scratch or bite. We report a patient with history of fever, swelling lymph nodes, vasculitic-like skin lesions, and positivity of *Bartonella* serology initially considered as expression of a disimmune disease.

## 1. Introduction

Cat scratch disease (CSD) is a bacterial disease caused by *Bartonella henselae*. It is mainly characterized by self-limiting lymphadenopathy (especially around the head, neck, and upper limbs) in the draining site of a cat scratch or bite. Other general symptoms include fever (usually less than 38°C), fatigue, loss of appetite, headache, rash, sore throat, and an overall ill feeling. In such cases, someone might have infections of the liver, spleen, heart, lungs, joints, bones, or a lingering high fever without other symptoms. Some get an eye infection (Parinaud oculoglandular syndrome), with symptoms including redness of the eye and swollen lymph nodes in front of the ear. Others may develop inflammation of the brain, although this is rare. All of these complications usually resolve without any lasting illness and the majority of mild-to-moderate cases of CSD resolve in 1 to 2 months without any antimicrobial therapy. The therapeutic approach varies on the basis of the clinical manifestations and immune status of the patient. There is a paucity of data in the literature as to the most effective therapy, with most data presented as part of case series rather than randomized controlled trials. There is a significant divide in the literature between *in vitro* efficacy of antibiotics and the ability to successfully treat in clinical practice. *In vitro*, *Bartonella* species have been found to be susceptible to a number of antimicrobial agents including macrolides, aminoglycosides, *β*-lactams, expanded-spectrum cephalosporins, trimethoprim-sulfamethoxazole, rifampin, and ciprofloxacin. Since the causative bacteria cannot be easily cultured from human lymph node samples, the diagnosis usually relies on epidemiological, clinical, histological, and serological criteria [1]. According to Bergmans et al. [2], a diagnosis of CSD usually requires three of the following four criteria: (I) a history of contact with a cat and the presence of a scratch or primary lesion of the skin, eye, or mucous membrane; (II) a positive cat scratch skin test reaction; (III) negative laboratory testing for other causes of lymphadenopathy; (IV) characteristic histopathological findings in a lymph node biopsy specimen or at a site of systemic involvement. However, none of these criteria are sufficiently specific to establish a diagnosis of CSD and to differentiate between several infectious etiologies. A typical CSD histology showing a granuloma with central necrosis, multinucleated giant cells, and microabscesses may also be absent. Thus, histological examination at an early stage of the disease in fact shows only lymphoid hyperplasia and arteriolar proliferation. Conversely, in the presence of granuloma, a differential diagnosis with respect to tuberculosis or other infectious diseases that display granulomas can be very difficult [1].

## 2. Case Presentation

A 58-year-old woman was admitted in our clinic with a history of recurrent necrotizing lymphadenopathy. In the personal history she disclosed that she was treated for hypothyroidism and she had an episode (2006) of vesiculobullous dermatitis which was diagnosed as contact eczema. She gave a negative history for any travel abroad and prior contact with pet.

She complained onset of painful left axillary lymph node package associated with fever (38°C) in February 2008. For that problem she was followed in the oncology department from 2008 to 2010. At the beginning of 2008 the first biopsy with histology suggestive but not diagnostic for Castelman's disease was made. For persistent symptoms a full-body CT scan was performed with detection of left axillary and supraclavicular lymph node package (8 × 6 cm with colliquative area), reactive lymph nodes (2 cm) in the right axilla (2 cm), inguinal lymph nodes (2 cm), nodules of uncertain origin (<1 cm) in the lung, and liver parenchyma. Repeated lymph node biopsy was not yet conclusive for diagnosis of Castleman's disease.

**Figure 1:**
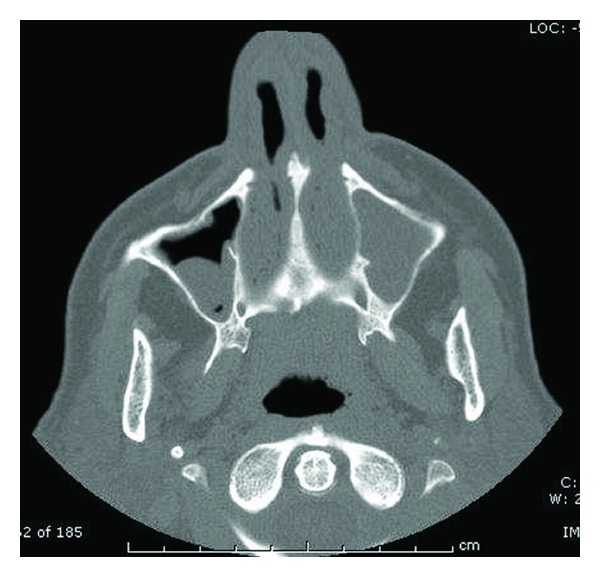
CT scan—mucous obliteration of the maxillary sinus (complete in the left, partial in the right); thickened and swollen nose soft tissues without osteostructural alterations.

**Figure 2:**
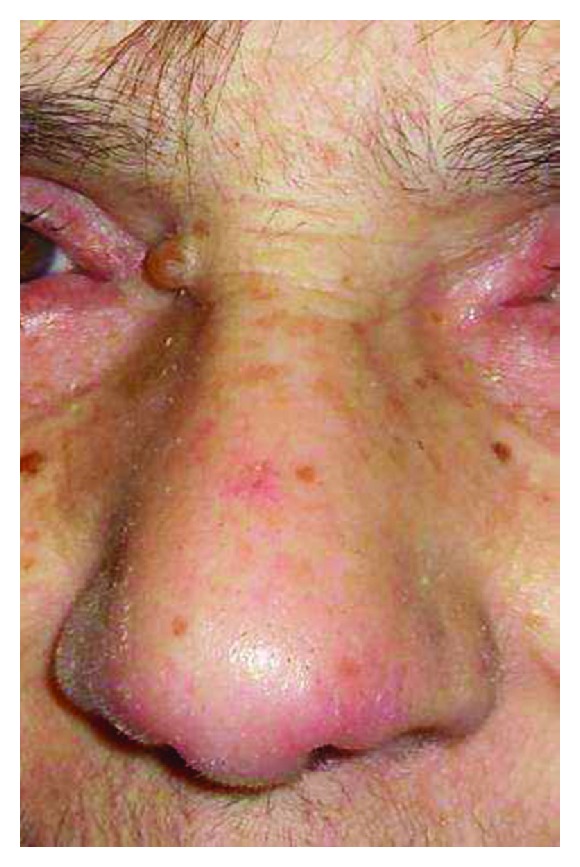
Swelling with features of chondritis and crusted lesions of the nose.

**Figure 3:**
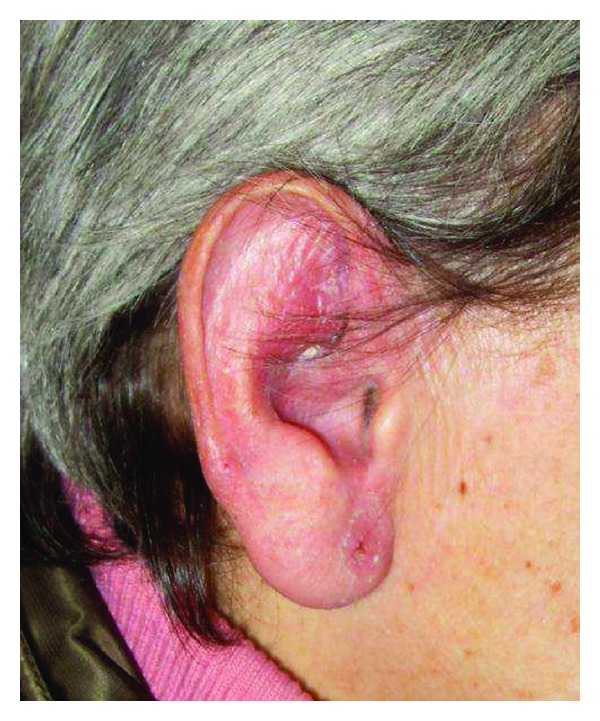
Swelling with features of chondritis and crusted lesions in the right ear.

**Figure 4:**
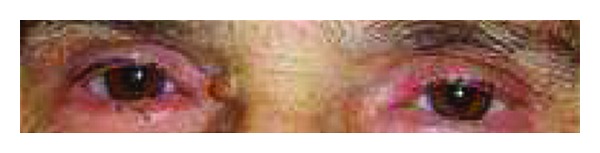
Chronic conjunctivitis with blepharitis aspects.

**Figure 5:**
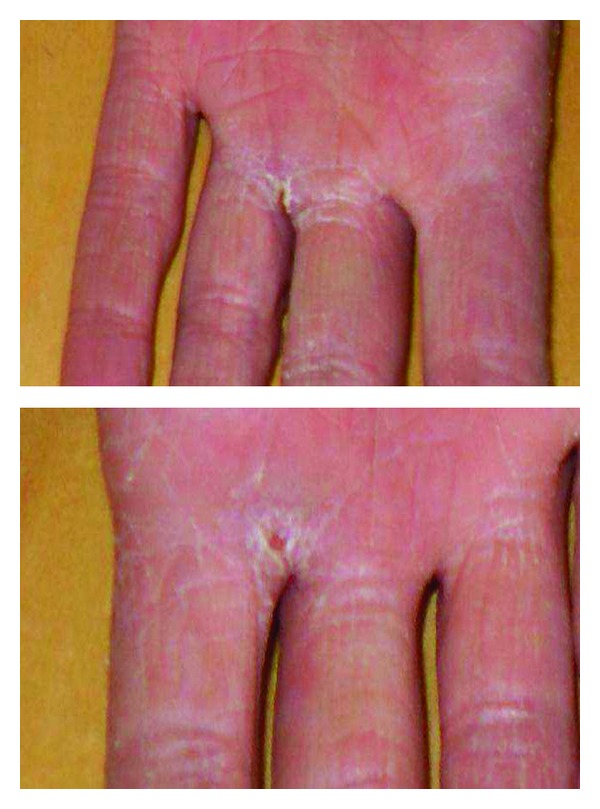
Vasculitic-like ulcerative lesions of the hands (palm).

For persistent fever inguinal biopsies were performed in June 2009 and February 2010 with confirmation of nonspecific necrotizing lymphadenitis. Microbiology and virology analysis were negative (in particular, HHV-6 and HHV-8). The full body CT scan of June 2010 showed right axillary and supraclavicular lymph nodes (up to 2.8 cm), no finding of colliquated lymph nodes previously described on the left, enlargement of inguinal lymphadenopathy bilaterally, right perianal mass (1.3 × 1.1 cm) similar to abscess, no more evidence of liver and lung lesions. On a CT scan performed for a sudden extremely painful nose swelling, a mucous obliteration of the maxillary sinus was revealed, nose soft tissues were thickened and swollen without osteostructural alterations (Figure 1). At the revision of previous histological biopsy samples, disclosure of necrotizing lymphadenitis with epithelioid/giant cells and vasculitis in the absence of lymphoproliferation signs was observed. These aspects were considered as not inconsistent with vasculitic disease. A first rheumatologic evaluation was carried out in January 2011. Physical examination revealed swelling with features of chondritis and crusted lesions in the nose and the right ear (Figures 2 and 3); chronic conjunctivitis with blepharitis aspects (Figure 4); multiple purple skin areas suggestive for vasculitic lesions in the upper and lower limbs, in particular ulcerative injuries of the hands (palm) (Figure 5) and ulcerative rhagadiform lesion in the gluteal region were disclosured as well as papular-crusted eruptions in the abdominal and umbilical area. Evening fever was persistent and relapsing. Palpable axillary and inguinal lymph nodes were painless, elastic, mobile; the remainder of thoracic and abdominal examination was unremarkable. In the suspicion of granulomatous vasculitis, nasal mucosa biopsy was performed. Histologic findings did not show specific aspects for Wegener's diagnosis: acute and chronic infiltrate was revealed but no evidence of vasculitis, necrosis or plurinucleate cells. Laboratory studies proved neutrophilic leukocytosis and elevated acute-phase reactants. Antineutrophil cytoplasmic antibodies (ANCA) were negative but anti-Beta 2 glycoprotein 1 autoantibodies IgG and IgM and anti-Cardiolipin autoantibodies IgG and IgM gave positive results. Among microbiology and virology analysis performed, IgM Cytomegalovirus-antibodies (with negative antigenemia) and IgM Epstein-Barr virus antibodies (with not significant PCR amplification) were found. *Bartonella *serology was also tested with disclosure of *anti-Bartonella henselae *levels corresponding to 1 : 128.

At the second rheumatologic evaluation the positivity of anti-Beta 2 glycoprotein 1 autoantibodies and anti-Cardiolipin autoantibodies was confirmed as well as *anti-Bartonella *levels (1 : 128). Considering persistent fever and skin lesions, in spite of steroid therapy previously adminisetred (prednisone 37.5 mg/day), infectivologic examination was required. Although *Bartonella *serology was considered aspecific finding as part of a more probable vasculitic disease, it was decided to administer antibiotic therapy in the suspicious of *Bartonella *lymphadenitis (Azitromicina 500 mg for 5 days). After the antimicrobial therapy (June 2011) lymphadenopathies, fever and vasculitic skin lesions gradually resolved. Given that vasculitic origin was still assumed, the patient was assigned to the rheumatologic followup to study the possible evolution of the disease. However, a persistent resolution of the previous clinical picture was observed at the evaluation of February 2012.

## 3. Discussion

Cat scratch disease caused by *B. henselae *is a relatively common and ubiquitous infectious disease, which has been recognized as one of the common causes of regional lymphadenopathy with an expanding spectrum of clinical manifestations. Detection and isolation of Bartonella species from arthropods, pets (usually cats but dogs too) and small wild animals is commonplace; this includes a variety of known and emerging Bartonella pathogens [3]. Laboratory diagnosis of CSD is now based on histologic findings, detection of antibodies to *B. henselae *in the serum, and PCR assays of tissue. All three *Bartonella *spp. (*B. henselae, B. quintana*, and *B. bacilliformis*) can induce cutaneous vasoproliferative lesions and bacteremia, whereas endocarditis and systemic vasoproliferative lesions with involvement of the brain, bone, bone marrow, lymph node, skeletal muscle, and mucosal surfaces are associated with *B. henselae *or *B. quintana *infections [4].

The Bartonella species have been recently recognized as important causative agents of culture-negative bacterial endocarditis. The majority of reported cases of *Bartonella *endocarditis affect native valves. Antineutrophil cytoplasmic antibodies (ANCAs) have been associated with the spectrum of idiopathic small vessel vasculitis. However, a variety of infections can result in a false-positive ANCA test, and especially subacute bacterial endocarditis (SBE) with the presence of ANCAs occasionally mimics the clinical manifestations of an ANCA-associated vasculitis such as skin purpura and glomerulonephritis. Therefore, it is crucial to distinguish an ANCA-positive SBE from an ANCA-associated vasculitis with endocardial compromise, because the misdiagnosis of an SBE as an ANCA-associated vasculitis can lead to an inappropriate immunosuppressive therapy with catastrophic consequences [5]. A review of renal biopsy findings in *B. henselae *native-valve endocarditis with associated glomerulonephritis (GN) revealed a pattern of immune complex-mediated GN with segmental necrotizing and crescentic lesions. ANCA-positive GN and vasculitic-like illness in patients with underlying endocarditis have been well described previously [6, 7]. Typical histological features of lymph nodes during CSD are inflammatory granulomas with microabscesses surrounded by histiocytes, occasional giant cells, lymphocytes, and fibrosis [8]. In *B. henselae *infection the histopathological presentations range from endothelial proliferations with interstitial accumulation of neutrophils, the characteristic “stellate microabscesses” of cat scratch lymphadenopathy, to hepatosplenic granulomas. The granulomatous reactions are mainly observed in immunocompetent patients. The endothelial dysfunction may be due to a direct cytopathic effect of *Bartonella *infection or due to soluble factors like vascular endothelial growth factor generated during the interaction of the bacteria with their host [9, 10].

In our patient the histology was that of necrotizing lymphadenitis with epithelioid/giant cells and aspects of vasculitis but no lymphoproliferation signs. These aspects were considered compatible with vasculitic disease and led to an initial presumptive diagnosis of Wegener's granulomatosis. However, antineutrophil cytoplasmic antibodies were negative and clinical picture was refractory to immunosuppressive therapy with steroids. *Bartonella *serology was incidentally tested and the positivity of anti-*Bartonella *was initially considered a nonspecific finding associated with a disimmune disease. However, the response to antimicrobial therapy was significant. Our patient did not have *Bartonella henselae* endocarditis which could have explained this vasculitis. Skin involvement, lymphadenopathy, and fever were the main features. As previously described in literature cutaneous vasculitis (hypersensitivity vasculitis) could disclosure cat scratch disease [11]. Therefore, in cases of vasculitic syndrome (with ulcerative skin involvement) lacking classical autoantibodies, infection with *B. henselae *should be considered, even without a history of contact with cats [4].
